# CDK8: a new breast cancer target

**DOI:** 10.18632/oncotarget.15354

**Published:** 2017-02-15

**Authors:** John Crown

**Affiliations:** Department of Medical Oncology, St Vincent's University Hospital, Dublin, Ireland

**Keywords:** CDK-8, breast cancer, estrogen receptor, CDK19, anti-estrogens

Close to 70% of all breast cancers express the estrogen receptor (ER), one of the oldest molecular targets in oncology. Once ER is bound to estrogen in the cytoplasm, it enters the nucleus where it binds to specific DNA sequences (estrogen response elements) in or near the promoters of a limited set of genes, inducing their transcription. ER-mediated transcription produces a mitogenic effect in ER-positive breast cancer cells. Agents interfering with ER signaling have long been used for hormone therapy of ER-positive breast cancers. These drugs include aromatase inhibitors that inhibit estrogen synthesis and selective estrogen receptor degraders (SERDs), which both block estrogen interaction with ER and cause ER degradation. In addition, selective estrogen receptor modifiers (SERMs, most notably tamoxifen) not only compete with estrogen for ER binding but also subvert the function of ER, changing its transcription-regulating activity in a way that suppresses rather than promotes the growth of breast cancers. While generally very efficient in an adjuvant setting, hormone therapy of metastatic breast cancer patients frequently fails, as their tumors modify their ER, making it less dependent on estrogen, or start utilizing other signal transduction pathways to replace ER. A recent article by McDermott et al. [1] from the laboratory of Eugenia Broude at the University of South Carolina suggests that a new class of experimental drugs targeting a transcriptional regulator CDK8 may have a positive impact on the challenges facing hormone therapy of ER-positive breast cancer.

CDK8 and its “twin” CDK19 belong to the same cyclin-dependent kinase (CDK) family as CDK4/6, the target of effective drugs that have recently entered the clinical armamentarium for ER-positive breast cancers. In contrast to CDK4/6, CDK8 does not mediate cell cycle progression and CDK8 inhibitors do not generally affect cell proliferation [2]. Instead, CDK8 acts as a co-regulator of transcription that cooperates with several transcription factors, in most cases enhancing the activity of these factors [3, 4]. CDK8 was found to be upregulated in breast cancers and associated with tumor progression; elevated expression of CDK8 and its interactive proteins has been linked to shorter relapse-free survival of breast cancer patients [2, 5, 6]. McDermott et al. [1] now found that CDK8 expression in breast cancers is inversely associated with the expression of ER, a finding that made them investigate if CDK8 could affect ER signaling. They showed that CDK8 inhibition, either with small-molecule CDK8 inhibitors (Senexin A and Senexin B) or by shRNA knockdown or CRISPR/CAS9 knockout of CDK8, suppresses both the transcriptional and the mitogenic effects of estrogen in ER-positive breast cancer cells. The effect of CDK8 is exerted downstream of ER, as the recruitment of CDK8 to estrogen-stimulated genes modifies RNA Polymerase II allowing it to complete transcription of ER-induced genes more effectively.

Senexin A and B inhibited the growth of ER-positive breast cancer cells and were synergistic with a SERD fulvestrant. On the other hand, combining CDK8 inhibitors with tamoxifen or other SERMs appears more problematic, since the SERMs rely on functional ER for their tumor-suppressive activity and CDK8 inhibitors, by inhibiting ER function, could interfere with this effect. McDermott et al. presented an *in vivo* study with an ER-positive breast cancer xenograft model, where Senexin B both inhibited the tumor growth alone and enhanced the tumor-suppressive activity of fulvestrant; these anti-tumor effects were mechanistically associated with the inhibition of an estrogen-regulated gene (GREB1) in the treated tumors. Notably, Senexin B alone and in combination with fulvestrant exerted therapeutic efficacy in this model without apparent toxicity or mouse body weight loss. These results are similar to the effects of another CDK8 inhibitor, Cortistatin A reported in leukemia models [7] but they differ from the significant toxicity that was recently reported for two other CDK8 inhibitors at doses that inhibited colon cancer xenograft growth [8]. These divergent preclinical results underscore the importance of selecting both the appropriate inhibitors and the most susceptible and biologically justified tumor types for the future clinical development of CDK8-targeting drugs.

Of special promise, CDK8 inhibitors suppressed the development of estrogen independence in different ER-positive breast cancer cell lines, when the cells were grown over a long term in estrogen-depleted media. This assay mimics the development of resistance to aromatase inhibitors and the results of McDermott et al. [1] suggest that CDK8 inhibitors may eventually be explored as the first line of therapy in combination with SERDs or aromatase inhibitors, both enhancing the efficacy and preventing the development of resistance to hormone therapy.
